# Cone-beam computed tomography analysis of primary root canals transportation and dentin loss after instrumentation with two-pediatric rotary files

**DOI:** 10.1186/s12903-022-02245-8

**Published:** 2022-05-31

**Authors:** Yasmine Ahmed Mortada Abd El fatah, Nagwa Mohamed Ali Khattab, Yasser Fathi Gomaa, Ahmad Abdel Hamid Elheeny

**Affiliations:** 1 Faculty of Dentistry, Assiut University, Assiut, Egypt; 2 Faculty of Dentistry, Ain Shams University, Cairo, Egypt; 3 Faculty of Dentistry, Minia University, El Minia, 61519 Egypt

**Keywords:** Child, Root canal, Cone-beam computed tomography, Nitinol

## Abstract

**Background:**

This in vitro study aims to compare rotary files (Fanta and Zuanba) with manual K files according to the amount of dentin removed and canal transportation in primary mandibular second molars by CBCT images.

**Methods:**

This experimental study was conducted on 60 extracted human second primary molars. That were divided into three groups according to root canal preparation group "I" instrumented with manual K-files, group "II" instrumented with rotary Fanta files, and group "III" instrumented with rotary Zuanba files. After root canal preparation, teeth were scanned before and after mechanical preparation with CBCT scanner. Then the amount of dentin removed was determined at three levels, including the coronal, middle and apical levels. Data were statically analysed using the Kruskal–Wallis test.

**Result:**

No statistically significant difference in the amount of dentin removed were noted between the manual and the rotary groups at the coronal and apical RC levels (P = 0.420) and (P = 0.819) respectively but significant difference was noted at the middle third (P = 0.043). Regarding RC transportation, no significant difference was noted among the three groups with the exception of the apical RC level (P = 0.043).

**Conclusion:**

Although no significant differences were noted between rotary and manual files, the rotary files showed better performance.

## Background

Deciduous dentition is important to a child's growth and development. It not only aids in mastication, speech, and aesthetics, but also maintains the space until permanent successors emerge. Crowding and malocclusion originate from the early loss of deciduous teeth and failure to preserve the space created [1].

Maintaining the arch's integrity requires maintaining deciduous teeth' vitality until their natural exfoliation period. Caries, restorative procedures, and trauma can all alter the pulp of primary dentition, which is histologically comparable to permanent teeth [2].

Root canal therapy (RCT) for deciduous teeth is the last option for preserving a deciduous tooth with pulp tissue that has been irreversibly damaged by caries or trauma in a child. Masticatory functions can be preserved, space for the succedaneous permanent teeth can be maintained, and early eruption can be avoided. The success of RCT is primarily determined by the reduction of germs and the prevention of reinfection [3, 4].

RCT can be performed using traditional or manual methods as well as rotary devices. Cleaning the root canal and removing permanently inflamed or necrotic pulp tissue are the first steps in root canal preparation followed by filling with a substance. The resorption period of the filling material should be the same as that of the primary tooth [5].

The canal was historically prepared with hand devices such as K files, reamers, and H files. Because most hand preparation procedures are time-consuming and have been found to generate iatrogenic problems (e.g., ledging, zipping, canal transportation, and apical obstruction), root canal preparation techniques using nickel titanium (Ni Ti) rotary devices have received much attention [6].

For both skilled and new operators, the advantages of rotating Ni–Ti equipment over manual preparation have been noted by a number of researchers. They discovered that using Ni–Ti rotary files for root canal preparation in primary teeth was less expensive and quicker and resulted in fillings that were dependably uniform and predictable [7, 8].

The clinical success of Profile, ProTaper, Mtwo, FlexMaster, Light Speed LSX, Hero 642, K3, and WaveOne rotary files in primary teeth has been noted by a number of authors. In the field of paediatric endodontics, rotary paedodontic files have made a significant breakthrough [9].

Several techniques have been used to evaluate the shaping ability of endodontic instruments including serial sectioning, radiographs, microscopic analyses, silicone impressions, muffle systems, endodontic cubes, multislice computed tomography, and CBCT. The CBCT is now commonly utilized for noninvasive assessment of root canals made with an endodontic instrument [10].

The aim of this study was to compare rotary files (Fanta and Zuanba) with manual K files according to the amount of dentin removed and RC transportation in primary mandibular second molars using CBCT. The null hypothesis (H_0_) supposed that there was no difference in the amount of dentin removed at the apical, middle, and cervical thirds of primary molars root canals after instrumentation with manual K-files and two recently introduced pediatric rotary files (Fanta and Zuanba).

## Methods

### Teeth selection, disinfection, storage and randomization

The Ethical committee of Faculty of Dentistry Minia University assessed the research protocol of present study (reference number 360/2019). All methods were carried out in accordance with CRIS (Checklist for Reporting In-vitro Studies) guidelines and regulations. Mesial roots of sixty human mandibular second primary molars with no history of endodontic treatment were collected from the outpatient clinic of the Pediatric Dentistry Department of the local university. Before launching the study, all teeth were inspected clinically and radiographically to ensure sample homogeneity. Only teeth with mild to moderate curvature angles based on Schneider's criteria [11] (mild angle of curvature equals ≤ 5° and moderate angle of curvature equals 10°–20°) and completed sound mesial roots were included. Teeth with internal root resorption and calcifications were excluded. Additionally, RCs with abnormal shapes (severely large or thin) or curvature were excluded. All specimens were washed under running water and all the soft tissue was removed from their crown and root surface with scalpel and gauze. Then, specimens immersed in 0.5% sodium hypochlorite for one week for disinfection, and stored in sterile saline water at 37 °C [12].

### Specimen randomization and allocation

An independent investigator (A.M.A) was responsible for the randomization process. To guarantee an equal distribution of specimens among the study groups, a computer-generated block randomization (https://www.sealedenvelope.com/simple-randomiser/v1/lists) was used (block size of 6). Teeth were assigned to three groups. Group "1" (control) was instrumented with hand K-files (MANI Inc., Tochigi, Japan), and groups "2" and "3" (intervention groups) were instrumented with rotary Fanta files (Fanta dental, China) and rotary Zuanba files (Zuanba, China) respectively.

### Pulpectomy procedures and mechanical instrumentation

For standardization all clinical procedures were performed by the same operator with 10 years of experience operating with rotary files. After removal of the decay and access of the pulp, a nonend cutting bur #558 under air/water coolant was used to clear the pulp chamber roof. Coronal pulp tissue remnants were removed using a sharp sterile excavator. For each tooth, the occlusal surface was reduced to a flattened surface to confirm similar reference points of all specimens. This process allowed a standardized of all instruments along the working lengths (WLs) of different samples. Mesial RCs were localized, and a handheld size 10 stainless steel K-file (MANI, Inc.; Tochigi, Japan) was inserted into the mesiobuccal RC until the file tip just emerged from the apical foramen. The WL was determined by subtracting one millimetre from the apical foramen [13].

For the control group, RCs were instrumented in a step back approach up to size 35 K-file using a balanced force technique. For the second group (Fanta intervention group), RC instrumentation was performed according to the recommended sequence by the manufacturer as follows: 17/0.08, 20/0.04, 25/0.04 and 30/0.04. The applied speed and torque were 350 rpm and 2 Ncm respectively. The RCs of the final group (Zuanba intervention group) were mechanically instrumented at a speed of 300 rpm and a torque of 2 Ncm. According to the manufacturer's guidelines, the adopted instrument sequences were 20/0.04, 25/0.04 and 30/0.04. All rotary instruments were rotated in 20:1 gear-reduction, torque controlled hand-piece powered by an X Smart Plus endomotor (Dentsply Maillefer, Ballaigues, Switzerland). A picking motion without pressure was applied during mechanical instrumentation of the two rotary systems. RCs in the rotary file groups were prepared adopting a crown-down technique. For all groups, the files were lubricated with 17% EDTA gel (Dolo®, Prevest DenPro, India). Between each instrument, RCs were irrigated with 5 mL 1% sodium hypochlorite. Irrigation was performed using 30-gauge side-vented needles (Endo-Top®, PPH CERKAMED, Stalowa Wola, Poland). The irrigation needle was calibrated to stop 2 mm from the WL with back-and-forth movements of 2–3 mm. Each RC was flushed with 5 ml normal saline and then dried with sterile paper points [13].

### Phantom preparation and scanning protocol

**Fig. 1:**
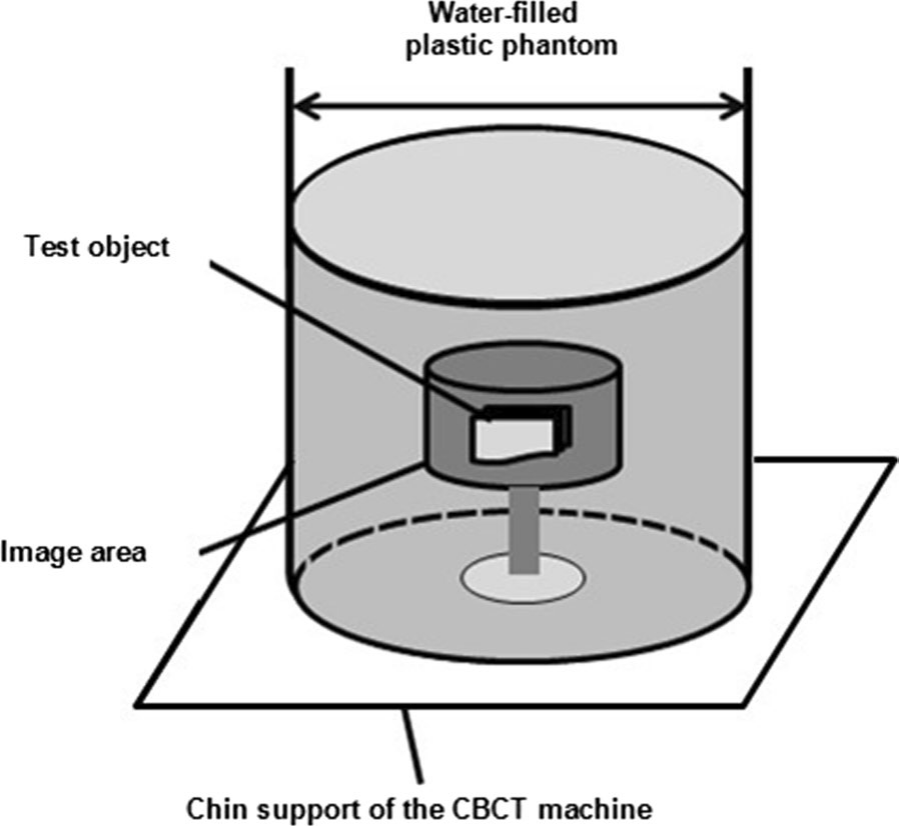
Phantom diagram which is a water-filled plastic cylinder containing water with dimensions of 150-mm diameter × 200-mm height

Each tooth was surrounded by wax and mounted in a block of silicon impression putty (Speedex, Coltene/Whaledent, Altstatten, Switzerland). Each sample was inserted with its long axis parallel to the long axis of the mold to ensure standardization of the specimens for CBCT imaging. The mold was mounted horizontally to fit the chin support of the CBCT machine. Each block was placed in a fine plastic cylinder containing water (a 150-mm diameter × 200-mm tall water-filled plastic cylinder was used as the head phantom to simulate soft tissue as illustrated in diagrammatic (Fig. 1) [14]. Pre- and postendodontic instrumentation, radiographic was performed using a CBCT scanner (SCANORA® 3Dx) with the following specifications: standardized kilo voltage = 90 kVP, 6 mA, 50 mm × 50 mm field of view (FOV) with 0.15 mm voxel sizes for high resolution, and a scanning time of 17 s.

### Quantitative assessment of root canal dentin thickness

**Fig. 2:**
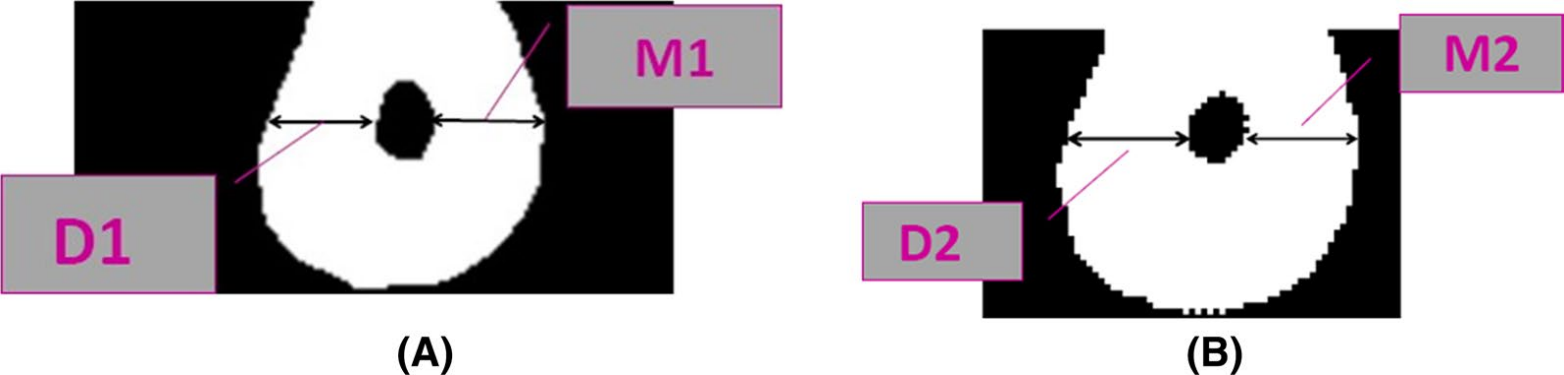
Quantitative measurements were defined: **A** the shortest distance between the outer surface of root mesial portion and the mesial wall of non-instrumented RC (M1) and the shortest distance between the outer surface of root distal portion and the distal wall of non-instrumented RC (D1), **B** the shortest distance between the outer surface of root mesial portion and the mesial wall of instrumented RC (M2) and the shortest distance between the outer surface of root distal portion and the distal wall of instrumented RC (D2)

Pre- and post-scanning CBCT images were completed for all specimens using similar exposure parameters. We examined the amount of dentin loss at three predetermined standardized reference points (1 mm, 3.5 mm, and 7 mm from the apex) representing the apical, middle, and coronal thirds of the RC, respectively. Quantitative measurement was performed to assess the dentin thickness before and after instrumentation. The following quantitative measurements were defined: the shortest distance between the outer surface of the root mesial portion and the mesial wall of the noninstrumented RC (M1), the shortest distance between the outer surface of the root mesial portion and the mesial wall of the instrumented RC (M2), the shortest distance between the outer surface of the root distal portion and the distal wall of the the noninstrumented RC (D1), and the shortest distance between the outer surface of the root distal portion and the distal wall of the instrumented RC (D2) [15] (Fig. 2).

### Root canal transportation

Predefined points M1, M2, D1, and D2 were used to determine the RC transportation. To calculate the RC transportation in the mesiodistal dimension, the following formula was used: (M1 − M2) − (D1 − D2) [16].

### Statistical analysis

For nonparametric data, the Kruskal–Wallis test was used to compare between the groups.

## Results

Regarding the changes in dentin thickness after instrumentation with the hand K-file and the two rotary systems, no statistically significant difference was found among the three groups at the cervical third (P = 0.420) and apical third (P = 0.819). In the middle third, the Fanta rotary system showed the lowest average amount of dentin removal postoperatively (mean of 0.069 ± 0.049 mm). The difference among groups at the middle third was statistically significant (P = 0.022) (Table 1).

**Table 1:** Amount of dentin removal mean and median values of the different file systems at coronal, middle, and apical levels

Root canal level	Zuanba (N = 20)	Fanta (N = 20)	Hand K-file (N = 20)	P*
Coronal				
Mean (SD) mm	0.107 (0.449)	0.166 (0.127)	0.189 (0.153)	0.420
Median (IQR) mm	0.115 (0.070)	0.125 (0.210)	0.125 (0.240)	
Middle				
Mean (SD) mm	0.103 (0.049)	0.069 (0.049)	0.115 (0.047)	**0.022**
Median (IQR) mm	0.080 (0.10)	0.080 (0.080)	0.130 (0.060)	
Apical				
Mean (SD) mm	0.085 (0.079)	0.085 (0.079)	0.036 (0.079)	0.819
Median (IQR) mm	0.050 (0.160)	0.050 (0.160)	0.045 (0.090)	

Bold indicate P-value set to ≤ 0.05

* Kruskal–Wallis test

**Fig. 3:**
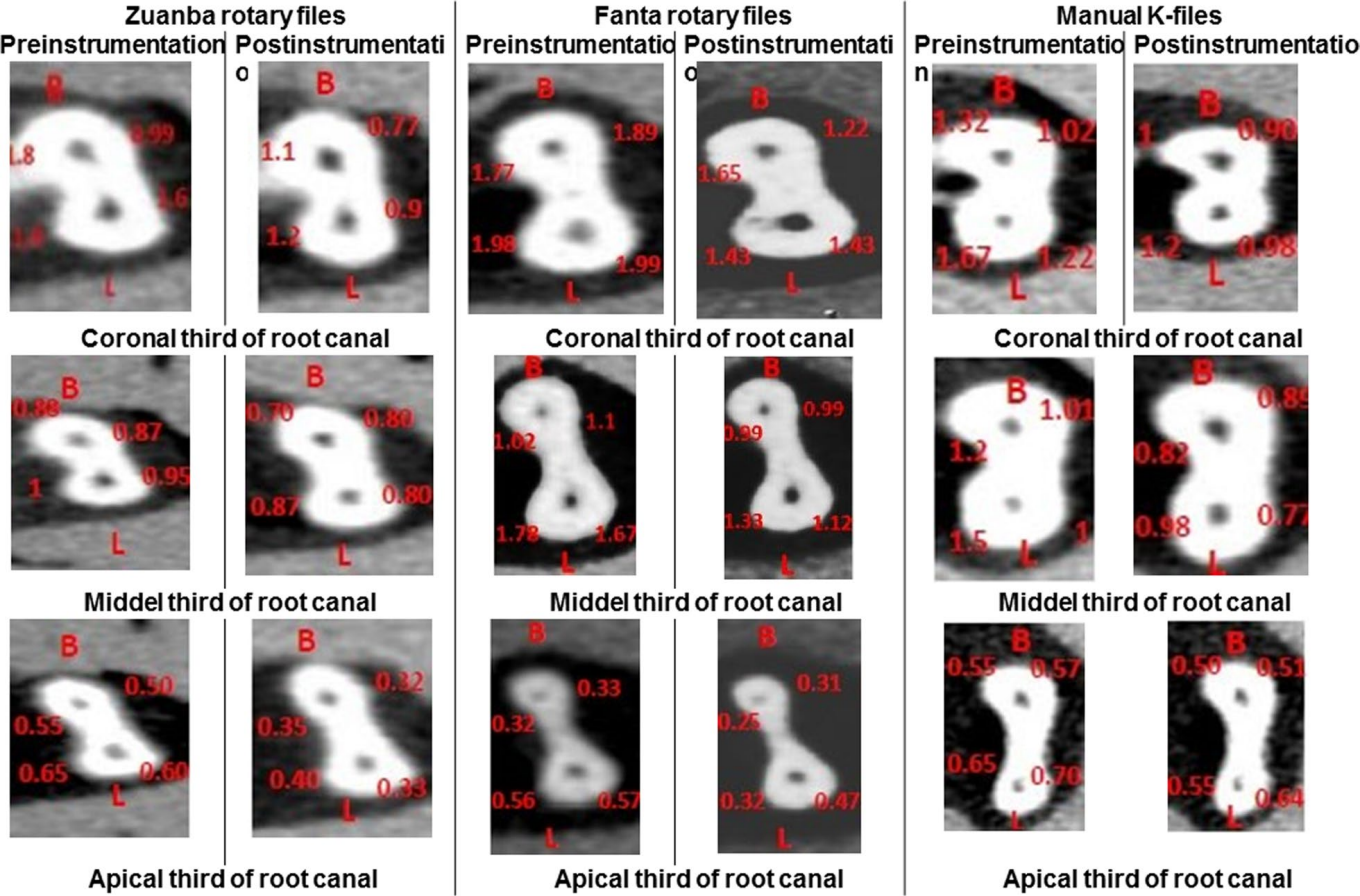
Pre- and post-instrumentation using Zunba rotary, Fanta rotary files, and manual K-files at coronal, middle, and apical thirds of the mandibular second primary molar mesial root canal

Data in Table 2 represent the average values of RC transportation of different study groups at different levels. In RC coronal and middle thirds, no statistically significant difference was detected between the control and intervention groups (P > 0.05). At the apical third of the RC, the RC mean transposition of the Fanta rotary (0.128 ± 0.070 mm) system was greater than the hand K-files (0.083 ± 0.069), and Zuanba rotary system (0.076 ± 0.072 mm). The difference between groups was statistically significant (P = 0.043) (Fig. 3).

**Table 2:** Mesial root canal transportation mean and median values of the different file systems at coronal, middle, and apical levels

Root canal level	Zuanba (N = 20)	Fanta (N = 20)	Hand K-file (N = 20)	P*
Coronal				
Mean (SD) mm	0.109 (0.074)	0.105 (0.056)	0.133 (0.055)	0.122
Median (IQR) mm	0.120 (0.130)	0.090 (0.080)	0.090 (0.080)	
Middle				
Mean (SD) mm	0.099 (0.040)	0.092 (0.051)	0.086 (0.044)	0.470
Median (IQR) mm	0.090 (0.050)	0.095 (0.080)	0.090 (0.050)	
Apical				
Mean (SD) mm	0.076 (0.072)	0.128 (0.070)	0.083 (0.069)	**0.043**
Median (IQR) mm	0.055 (0.080)	0.115 (0.10)	0.055 (0.080)	

Bold indicate P-value set to ≤ 0.05

*Kruskal–Wallis test

## Discussion

The use of specialized pediatric rotary files in pediatric endodontics has changed pulpectomy in the primary preparation of the primary tooth's canal [17]. The purpose of this in vitro study was to compare rotary pediatric files (Fanta and Zuanba) with manual K files using CBCT based on the amount of dentin removal and RC transportation in primary mandibular second molars.

The use of resin blocks and extracted natural teeth is a more widespread option among the options available for evaluating canal preparation. Extracted natural teeth were used in this study because they can almost perfectly simulate the microenvironment of root canal preparation in a clinical setting. However, the standardization of the apical patency of teeth, the compatibility of the apex with a specified instrument size, and the angle of curvature are all major limitations of their use in studies [18].

Teeth were selected based on at least two-thirds of root length as the inclusion criterion. The rationale behind this criterion was to assess the cleaning and shaping ability of different root canal instrumentation techniques until the apical third, where the maximum amount of bacteria is present. The primary teeth are always under a constant stage of dynamism. Two-thirds of the working length of the root canal is essential for the standardization of specimens. Numerous studies have used the same inclusion criterion [19–21].

In the current study, mesial root canals were prepared to maintain the homogeneity of the preparation made with both techniques given that the distal roots in primary mandibular molars show maximum variation in the occurrence of one versus two canals [22]. The root canal preparations were all completed by the same dentist. As a result, the operator was not considered a variable [23].

To date, a variety of approaches have been employed to assess canal form before and after instrumentation [19]. Plastic models, histologic sections, serial sectioning, scanning electron microscopic examinations, radiographic comparison, and silicone impression of non-instrumented canals have all been used to evaluate endodontic instrumentation [24].

In this study, the root canal anatomy and preparation were assessed by CBCT. The CBCT allows for much faster image gathering and reconstruction, resulting in higher quality and more precise images of the canals.

This system enabled dynamic visualization and assessment of specimens before and after instrumentation using predetermined standards, all without the need for examiner intervention [25]. Many authors have evaluated canal morphology in permanent teeth with CBCT demonstrating its effectiveness for analysing canal architecture [26, 27]. Similarly, this study also shows CBCT as an effective tool for the evaluation of canal morphology in primary teeth [15, 28].

To our knowledge, this is the first study that evaluated and compared the shaping abilities of these file (Fanta and Zuanba) systems in primary teeth. These rotary files were selected because they represent new trends in pediatric files with short lengths of 16 mm.

The amount of dentin removed reflects the instrument's aggressiveness. Especially in primary teeth, when the remaining dentin thickness is reduced following root canal instrumentation, the tooth exfoliates more quickly [29].

The results of the current study were partially accepted the null hypothesis as there was no statistically significant differences in the amount of dentin removed between the manual and rotary groups at the coronal and apical root canal levels. While, significant difference at the middle level among the three groups was found (null hypothesis was rejected). These findings may be attributed to the difference in the file design itself. According to the manufacturer, Fanta files are controlled memory (CM) files in which CM wires are manufactured by a proprietary thermomechanical process, which allows the instruments to be precurved before they are placed into the root canals. These files tend to adapt to the canal morphology and do not fully straighten during preparation of curved canals. In addition, this process also increases the flexibility, reduces the shape memory, and helps in obtaining stable martensitic at body temperature. The file design is a triangular cross section and advanced tip process that avoids forming steps. The file is a 16-mm length design and has improved resistance to cyclic fatigue, providing safer experience. Additionally, Zuanba blue rotary files are newly developed CM wires combined with a titanium oxide surface treatment, allowing for better flexibility, hardness and resistance to fracture. This feature allows the files to follow even the most tortuous canals with minimal risk of perforations or ledges. The blue file is flexible, tough, and precurved and suitable for bending curved root canals. The file is 16 mm in length.

K-files are stainless steel wire files with stiffer cross-sections through which they are able to press laterally against dentinal walls and result in efficient debridement [30].

The previous results are consistent with the results reported by Prabhakar et al. and Seema et al., who found no significant difference in cleaning efficiency between manual and other rotary systems when used in primary teeth [19, 29]. Other deciduous molar studies comparing manual files and rotary instrumentation found significant differences in the amount of dentine removal. For example, Kummer et al., Selvakumar et al., and Musale et al. found that rotary systems exhibited superior overall cleanliness than manual systems. The degree of root canal curvature, the number of files, instrumentation procedures, irrigation protocols, and cleaning evaluation methods may all play a role in this difference [15, 31, 32].

The prognosis may be affected by transportation after root canal shaping procedures, non-central root canal preparation, and insufficient or excessive instrumentation of the tooth structure. As a result, determining the quality of root canal preparation is critical for choosing the correct file system [33]. As a result, canal transportation was measured in the current study as the variance in the amount of dentin removed from canals at three different locations: apical, middle, and coronal from the apex. The file system with the least amount of canal curvature transportation in these defined positions was thought to have higher shaping ability and better preservation of the original canal morphology [34].

Considering the direction of canal transportation, all groups showed a tendency towards transport to the mesial (outer) direction, which is considered a safe area. Although all the systems tested in the present study produced some degree of canal transportation with no statistically significant differences at the coronal and middle root canal levels, statistically significant differences at the apical level were noted the three groups. This finding may be because primary teeth are always in a dynamic stage of resorption, which subsequently results in softer dentin at the apex. Therefore, equal shaping of the canal is achieved, and less transportation towards the curvature is noted. Another possible explanation is that the center of the preparation shifts in a clockwise direction with continuous rotation [35].

From clinical perspective, the use of pediatric rotary files is significantly improves the quality of obturation compared to the manual files. This could be related to the taper shape preparation (i.e. funnel shaped) produced by the rotary file which allows better loading of the filling material [36]. Additionally, pediatric rotary files influence the child's behavior positively as enhancing children's cooperation potential [37]. Other merits were reported including lesser instrumentation time and lower postoperative pain due to the lesser amount of periapically extruded debris which triggers inflammatory process compared to those accounted for the manual files [38].

Another important point regarding the use of pediatric rotary files with taper of 0.04 as those adopted in the current study diminishes the risk of root perforations compared to rotary files with tapers of 0.06 and 0.08 [39]. The higher the taper of rotary files, the increased the amount of dentin elimination from the root canal wall and subsequently the risk of root fragility increased [40]. This was consistent with the findings of Zameer et al. who found no significant difference between taper of 0.02 and 0.04 in dentin removal without endangering the root walls and at the same time attaining adequate shaping of the root canal [41]. Moreover, Nazari Moghaddam et al., reported safer primary root canal mechanical preparation with continuous rotation kinetics up to size 30 without excessive elimination of dentin [42].

### Study strengths and limitations

Although we adopted rigorous measures to achieve a maximum level of standardization such as identical tooth preparation performed with a single investigator and the use of CBCT technique which allows 3D quantitative analysis of amount of dentin thickness before and after mechanical instrumentation. Some concerns have to be taken into account. For instance, the in vitro trial nature of the trial is not typically mimics the oral environment, and subsequently complete control over the parameters can't be achieved. Thus, in-vivo experimental clinical trials are required that may provide new insight into the desirable marginal gap values in pediatric patients.

## Conclusions

The following conclusions can be drawn based on the study's limitations:

1. Except in the middle third, no statistically significant differences in dentin thickness were noted before and after canal preparation.
2. Except at the apical level, no significant difference in canal transportation was noted between the manual and rotary groups at the RC level.

## Data Availability

The datasets used during the current study available from the corresponding author on reasonable request. All data analyzed during this study are included in this published article in the form of tables and figures.

## Abbreviations

CBCT: Cone-beam computed tomography
RCT: Root canal treatment
RC: Root canal
WLs: Working length
CM: Controlled memory
